# Omega-3 Supplementation in Coronary Artery Bypass Graft Patients: Impact on ICU Stay and Hospital Stay—A Systematic Review and Meta-Analysis

**DOI:** 10.3390/nu16193298

**Published:** 2024-09-29

**Authors:** Asma Ouagueni, Zumin Shi, Mujahed Shraim, Raed M. Al-Zoubi, Ahmad Zarour, Abdulla Al-Ansari, Hiba Bawadi

**Affiliations:** 1 Department of Human Nutrition, College of Health Science, QU-Health, Qatar University, Doha 2713, Qatar; ao1601621@qu.edu.qa (A.O.); zumin@qu.edu.qa (Z.S.); 2 Department of Public Health, College of Health Science, QU-Health, Qatar University, Doha 2713, Qatar; mshraim@qu.edu.qa; 3 Surgical Research Section, Department of Surgery, Hamad Medical Corporation, Doha 576214, Qatar; ralzoubi@hamad.qa (R.M.A.-Z.); alansari1@hamad.qa (A.A.-A.); 4 Department of Chemistry, Jordan University of Science and Technology, P.O. Box 3030, Irbid 22110, Jordan; 5 Department of Biomedical Sciences, College of Health Science, Qatar University, Doha 2713, Qatar; 6 Acute Care Surgery Division, Department of Surgery, Hamad Medical Corporation, Doha 576214, Qatar; azarour@hamad.qa; 7 Department of Surgery, Division of Urology/Andrology, Hamad Medical Corporation, Doha 576214, Qatar

**Keywords:** CABG, ICU, hospital stay, omega-3 PUFA, meta-analysis

## Abstract

**Background/Objectives**: Coronary artery bypass graft (CABG) is associated with inflammation and complications, potentially leading to prolonged ICU and hospital stays. Omega-3 PUFA has anti-inflammatory properties, thought to potentially reduce complications in CABG patients. This study aims to systematically review and meta-analyze the impact of perioperative omega-3 PUFA supplementation on total ICU and total hospital stays in CABG patients; **Methods**: Randomized controlled trials examining the effects of omega-3 PUFA supplementation (IV/oral) on ICU and hospital stays in CABG patients were included. Studies were searched for in PubMed, EMBASE, PsychINFO, CINAHL, and the Cochrane Central Register of Controlled Trial databases, along with hand searching of reference lists. The quality and risk of bias of the included studies were evaluated by two independent reviewers using the revised Cochrane risk-of-bias tool. Meta-analysis was performed using fixed or random effects models according to the level of heterogeneity by mean difference with their 95% confidence intervals; **Results**: Twelve studies were included in the qualitative analysis and seven in the meta-analysis. Omega-3 PUFA was associated with a significant reduction in days of hospital stay (−0.58 (95% CI −1.13, −0.04)). Subgroup analysis showed that only oral omega-3 PUFA supplementation resulted in a statistically significant reduction in length of hospitalization after subgroup analysis with MD −0.6 (95% CI −1.17, −0.04); **Conclusions**: This study suggests that perioperative omega-3 PUFA supplementation may reduce the length of hospitalization in CABG patients, especially when administered orally. However, the findings should be interpreted cautiously due to the high level of heterogeneity.

## 1. Introduction

Coronary artery disease is one of the leading causes of mortality and morbidity worldwide [1]; therefore, it is managed by multiple procedures, including allowing the patient to undergo CABG surgery where an alternate route (graft) is connected before and after the obstruction, thereby allowing the blood to bypass it and renourish the blood flow to the affected areas. Coronary artery bypass graft (CABG), or heart bypass surgery, is the most common cardiac surgery procedure in the world as the average incidence rate is estimated to be 62 per 100,000 persons in Europe and 200,000 cases per year in the United States [2,3]. CABG surgery is a medical procedure to improve obstructive coronary artery disease (CAD) [4]. CABG surgery is known to cause inflammation in the body [5,6,7,8] and multiple post-surgical complications including intracranial bleeding [9], re-operation [10], and acute kidney injury [11,12], leading to increasing length of ICU stay and length of hospitalization post-surgery.

Many factors are associated with developing coronary artery disease including smoking [13], obesity [14,15], and poor diet [16]. Lifestyle factors play a crucial role in the development of CAD, and healthy lifestyle interventions such as diet can effectively reduce the risk of the disease [17,18]. Following a Mediterranean diet [19], healthy eating index [20], and dietary approaches to stop hypertension (DASH) diets [21] are suggested to protect against heart diseases. What these diets have in common are that they are rich in omega-3 polyunsaturated fatty acids (PUFAs). Omega-3 PUFAs are a group of polyunsaturated fatty acids that are obtained from marine and plant sources [22,23]. Three types of omega-3 that humans can obtain from the diet are docosahexaenoic acid (DHA), eicosatetraenoic acid (EPA), and their precursor, alpha-linolenic acid (ALA) [24]. In clinical settings, the enrichment of parenteral nutrition (PN) with omega-3s in perioperative status has adverse advantages in terms of length of hospitalization, ICU admission, and reduction in infection rates [25]. These improvements are due to the anti-inflammatory properties of omega-3 PUFAs [26,27].

The effect of perioperative supplementation of omega-3 PUFA on open heart surgeries including coronary artery bypass graft surgeries has been assessed in the literature by several clinical trials and systematic reviews. It is found to improve post-surgical outcomes including post-operative arterial fibrillation (POAF) in open heart surgeries and reducing the risk of mortality post-operatively [28,29]. The effect of omega-3 PUFA on post-operative atrial fibrillation has been studied extensively and many systematic reviews were conducted in the literature [30,31,32,33]. However, other important clinical outcomes such as length of ICU stay and length of hospitalization post-CABG surgery were not studied extensively; two systematic reviews conducted in 2017 looked at the effect on all open heart surgeries and the exposures were not limited to only omega-3 PUFA [34,35]. To our knowledge, no systematic reviews have summarized the literature on the effect of only perioperative omega-3 PUFA for patients undergoing mainly CABG surgery and the impact on ICU stay and length of hospitalization. This paper aims to systematically review and meta-analyze the findings of randomized controlled trials assessing the impact of perioperative omega-3 PUFA administration for patients undergoing CABG surgery and the effect on ICU stay and length of hospitalization.

## 2. Materials and Methods

The current systematic review and meta-analysis were conducted following the Preferred Reporting Items for Systematic Reviews and Meta-Analyses (PRISMA) guidelines [36], checklist is provided in Table S1. It is registered in the International Prospective Register of Systematic Reviews (PROSPERO) with the protocol number CRD42023318725.

### 2.1. Search Strategy

A comprehensive literature search strategy was conducted on five databases which are Medline (Pubmed), EMBASE, PsychINFO, CINAHL, and the Cochrane Central Register of Controlled Trial databases. The Health Databases Advanced Search (HDAS) [37] developed by the National Institute for Health and Care Excellence Databases search was used in collecting articles from the previously mentioned databases. Furthermore, hand searching of the supplementary search including reference lists of primary studies, review papers, and previous systematic reviews and meta-analyses was performed.

The search initially covered papers up to the 12th of September 2023 to ensure an extensive and comprehensive search to include all the recently published papers reporting on the effects of omega-3 PUFAs on ICU stay and length of hospital stay in patients undergoing CABG surgery. The search included all the concepts of population (CABG), intervention (Omega-3 PUFA), control (usual care/placebo), and outcomes (total ICU stay and total hospital length of stay). Appropriate controlled vocabularies or syntax were used in search strategies (e.g., MeSH in Pubmed, EMTREE in EMBASE) and free-text terms retrieved through previous systematic reviews search strategy and extra hand search of synonyms were carried out. Boolean operators “OR” and “AND” were used in creating the search strategy where the former was used for combining synonyms within the same concept and the latter for combining different concepts. The following are examples of keywords employed in the search: “Omega-3” OR “Omega 3” OR “Fish oil” OR “Eicosapentaenoic Acid” OR “EicosapentaenoicAcid” OR “EPA” OR “Docosahexaenoic acid*” OR “Docosahexenoic acid*” in all-fields keywords AND “exp *Coronary artery bypass/” OR “aortocoronary Bypass” OR “coronary artery bypass” OR CABG in all-fields keywords. Additionally, we used the keywords “intensive care unit”/exp OR “close attention unit” OR “combined medical and surgical icu” OR “length of hospital stay” OR “hospitalization”/exp OR “hospital stay”. No filters of language, publication year, publication type, or publication status were applied when searching the keywords to ensure the collection of all possible research papers. Details on the search strategy used in the databases are included in Table S2 and an example of a detailed search strategy in one database (EMBASE) is provided in Table S3.

#### 2.1.1. Eligibility Criteria

To structure the eligibility criteria, the PICO/TS framework (Patient/Population; Intervention; Comparison; Outcome; Timing; Setting/Study design) was used. Included studies were as follows: Population: adult patients (≥18 years old) undergoing mainly coronary artery bypass graft (CABG) surgery; Intervention: only long-chain omega-3 polyunsaturated fatty acids (PUFAs) containing both EPA and DHA molecules; Time: provided in duration starting from the pre-operative until the post-operative period. The method of administration was open to either oral supplementation or intravenous administration; Control: usual care or other non-fish oils; Outcomes: length of intensive care unit (ICU) stay and length of hospitalization post-CABG surgery; Setting/Study design: randomized controlled trials (RCTs). The excluded studies were as follows: all non-randomized trials, studies published in languages other than English, performed on other populations, e.g., children, used a combination of exposures with omega-3 such as vitamins and antioxidants cocktails, duration of supplementation was either pre-operative or post-operative only, and looked at outcomes other than hospital length of stay and ICU stay. Furthermore, we excluded studies that investigated the association of other open heart surgeries but did not include CABG, with omega-3 or fish oils.

#### 2.1.2. Selection of the Studies

The extracted studies were transferred to the Rayyan platform [38]. Duplicates were also removed using the Rayyan platform prior to screening. The screening was performed in two phases. The first phase was screening only titles and abstracts of the retrieved papers, and then the included papers were further screened for the full text following the inclusion criteria. The whole screening phase was performed by two independent reviewers to identify articles potentially eligible for inclusion in the systematic review. The reason for exclusion was documented in case of the exclusion of any study. The full texts of the screened studies were then critically reviewed separately for eligibility and data extraction. Any discrepancy in evaluation between the reviewers was resolved through meetings and discussions.

### 2.2. Data Extraction

Data from each study were extracted by one reviewer and revised by another reviewer in Microsoft Excel^®^ version 16.89.1 form and categorized as follows: first author, year of publication, sample size, number of patients in each study arm (intervention and control), type of trial (double-blind or open-label RCT), control used, omega-3 dose, method of administration (oral or intravenous), duration of supplementation, and follow-up duration. Outcomes and effect estimates from the intention to treat the outcomes of interest were extracted. The authors were contacted to obtain missing and unclear information.

### 2.3. Assessment of Articles Quality

To assure the quality, reliability, and validity of the included studies and to have insight into what extent the results concluded in this systematic review and meta-analysis apply to real-world scenarios, internal validity through risk-of-bias assessment was performed. Since the aim of this systematic review is to collect only randomized controlled trials addressing the research question, we used the revised Cochrane’s risk-of-bias tool for randomized trials (RoB 2) [39]. During the assessment of the quality of the studies, each study was independently assessed by two reviewers, and conflicts were solved by meeting with a third reviewer. The completeness of reporting was assessed by checking if the studies had reported all of the outcomes specified in their protocols. Trial registries were also searched in the Cochrane Central Register of Controlled Trial databases. Furthermore, an assessment of external validity in terms of generalizability and applicability source of funding and the presence of conflict of interest was performed.

### 2.4. Statistical Analysis

Since the outcomes in all studies were reported as continuous variables, the mean difference (MD) was calculated. The mean difference (MD) and 95% CIs were used as the effect size (ES) measure. All results were calculated on an intention-to-treat basis. I^2^ statistics were used to test for heterogeneity between studies [28]. A fixed and random effects meta-analysis was used based on the level of heterogeneity of studies. The pooled therapy effect estimate was measured as a weighted average of the therapy effects MD < 0 favoring omega-3 over the control. In studies reporting the outcomes as median and interquartile ranges (IQRs), validated conversion equations were used to calculate mean and SD, and they were considered for inclusion in the meta-analysis if the data were normally distributed [40]. Forest plots were used to summarize the clinical endpoints of interest for each study. The presence of publication bias was assessed through the visual inspection of the funnel plot and the Egger bias test [41] when sufficient papers were collected for the outcome. If there was no publication bias, the funnel plot would look like a symmetrical and inverted funnel, with a few studies scattered at the bottom. To assess the robustness of the results of the meta-analysis, a sensitivity analysis was conducted by using different statistical models. All analyses were performed using STATA SE software version 17 (STATA Corporation, College Station, TX, USA).

## 3. Results

### 3.1. Search Outcome

The comprehensive literature search resulted in a total of 878 records, where 871 citations were retrieved from the databases and 7 additional search results were identified through the supplementary search of a reference list of included studies, reviews, and previously published systematic reviews.

**Figure 1:**
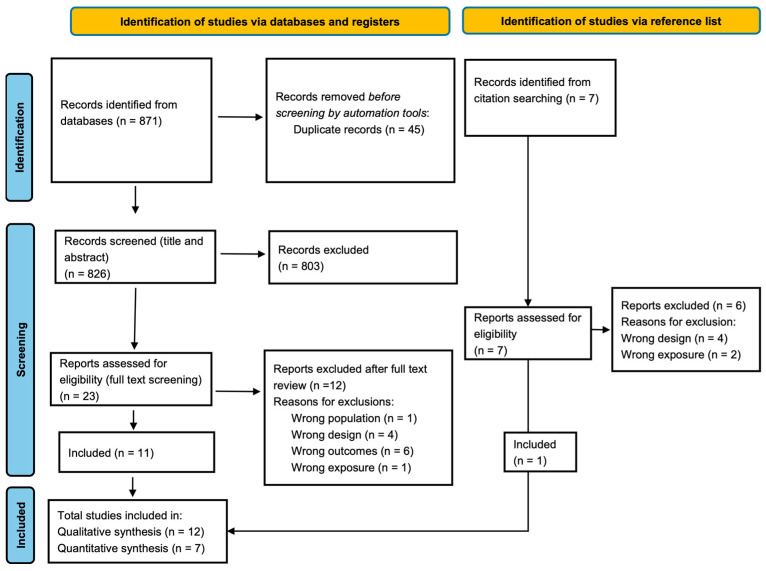
Study flow diagram based on the PRISMA checklist.

The 871 citations were retrieved from four databases: Medline (PubMed), EMBASE, CINAHL, and the Cochrane Central Register of Controlled Trial Library. Before the screening, 45 duplicates were identified and thereby removed, which then resulted in a total of 826. Further, 803 results were then excluded after screening titles and abstracts. A total number of 23 papers were consequently selected for full text article screening and were assessed for eligibility. However, 12 records were excluded due to several reasons: different population (n = 1), different study design (n = 4), outcomes other than the study aim (n = 6), and different exposure (n = 1). A table of reasons for exclusions is provided in Table S4. Furthermore, hand searching of the supplementary search including reference lists of primary studies, review papers, and previous systematic reviews and meta-analyses was carried out to ensure a comprehensive literature search strategy. This yielded a further 7 studies, and 6 of them were excluded because they did not meet the research question due to different study designs (n = 4) and different exposures (n = 2). A table of reasons for exclusions after full text is provided in Table S5. Only one study ultimately met the inclusion criteria for the qualitative synthesis. This ended up having a total of 12 papers included in the qualitative analysis, while the quantitative analysis was performed on 7 articles. Figure 1 shows the study flow diagram based on the PRISMA checklist.

### 3.2. Characteristics of Included Studies

A total of 12 studies met the eligibility criteria for inclusion with a total sample size of (n = 3569) participants. Table 1 shows the characteristics of the included studies in the systematic review. The studies included varied in terms of key characteristics. Variation was observed in study design, sample size, types of surgeries, sample characteristics, omega-3 PUFA dose, EPA/DHA ratio, route of administration, and duration of intervention.

The majority of the studies found on omega-3 PUFA related to intensive care unit stay and length of hospitalization after CABG surgery were double-blinded RCTs [42,43,44,45,46,47,48,49,50,51], except for two RCTs, one of which was open-label [52], while the second was a single-blinded study [29]. In terms of geographic distribution, the studies included were conducted across several regions. Specifically, two studies took place in Germany [29,42], two in Italy [49,52], and an additional two in the United States [47,48]. The remainder of the studies encompassed a variety of locations, including the United Kingdom [44], Australia [46], Iran [50], Mexico [51], and Russia [43]. One large study was conducted in 28 centers across Italy, the United States, and Argentina [45]. The sample size varied between 28 and 1516 participants among the different study designs. Due to the nature of the surgery, the participants were older adults and the elderly, and the mean age was above 58 years. Most studies had male participants as the majority in both study arms, one had included only males in their study [51], and another one included only males in the intervention group [29]. Additionally, there were some discrepancies in the type of surgery, and some assessed the effect of omega-3 supplementation on patients undergoing only CABG surgery [42,50,51,52]. One study specified the type of CABG and restricted inclusion to patients undergoing CABG by cardiopulmonary bypass (CPB) or “on-pump CABG” [29,43,44]. Another study compared the impact of CABG operative techniques “on-pump” or “off-pump” in addition to omega-3 supplementation on surgical outcomes [49]. Four studies included patients undergoing CABG, valve replacement, valve repair, or any combination of these surgeries, with the majority of participants undergoing CABG surgery [29,45,46,47,48]. The majority of studies used oral routes for perioperative omega-3 PUFA administration [44,45,46,47,48,49,50,51,52], while only three addressed the effect of short-term perioperative intravenous administration of omega-3 [29,42,43]. Regarding the studied outcomes, ICU length of stay was assessed by nine studies while total hospital length of stay was assessed by eleven papers. None of the studies included ICU or hospital length of stay as the primary outcome.

**Table 1:** Characteristics of included studies in the systematic review.

Study	Country	Design	Surgery	n (I/C)	Age (Mean ± SD)	Men (%)	Dose	EPA/DHA Ratio	Duration of Supplementation	Length of Hospital Stay Length of ICU stay
Berger, M.M., 2013 [29]	Germany	RCT, SB	CABG ± valve surgery (CPB)	I: 14	64.7 ± 10.5	14 (100%)	400 mg FO/kg over 2 infusions of 200 mg FO/kg at 12 h + 2 h Pre-OD 200 mg FO/kg Post-OD	Not specified	Pre-OD 1 to Post-OD 1	(=) ICU stay (*p* = 0.118)
				C: 14	66.3 ± 9.5	11 (78.5%)	Saline			
Heidt, M.C., 2009 [42]	Germany	RCT, DB	CABG	I: 52	M: 61.2 ± 14.1 F: 74.4 ± 9.2	38 (73%)	100 mg FO/kg/day	Not specified	12 h Pre-OD to ICU discharge	Comparison between pts with AF to without AF (variance analysis) (=) ICU stays (*p* > 0.05) * (=) Hospital stays (*p* > 0.05) *
				C: 50	M: 66.6 ±10.7 F: 70.7 ± 8.2	32 (64%)	FFA 100 mg Soya oil/kg/day containing 10 g Soya oil			
Lomivorotov, V.V., 2014 [43]	Russia	RCT, DB	CABG (CPB)	I: 18	61	17 (94.4%)	Pre-OD 1: 200 mg/kg/day Post-OD 2–7: 100 mg/kg/day	Not specified	Pre-OD 1 to 2–7 Post-OD	(=) ICU stay (*p* = 0.97) (=) Hospital Stay (*p* = 0.56)
				C: 21	58	20 (95%)	Pre-OD 1: 2 mL/kg/day Post-OD 2–7: 1 mL/kg/day Intralipid			
Calò, 2005 [52]	Italy	OL, RCT, Parallel groups	CABG	I: 81	64.9 ± 9.1	68 (84%)	1.7 g/day 850 mg EPA, 882 mg DHA	1:2	Pre-OD 5 to discharge	(-) length of hospital stays after surgery (*p* = 0.017)
				C: 79	66.2 ± 8.0	68 (84%)	Usual care			
Saravanan, P., 2009 [44]	UK	SC, RCT, DB	CABG (CPB)	I: 52	64 ± 11	40 (77%)	2 g/day	1.2:1	17 days (median) Pre-OD 12 to 21 until discharge	(=) Hospital stay (*p* = 0.49) (=) ICU stay (*p* > 0.05) *
				C: 51	64 ± 10	42 (82%)	2 g/day Olive oil		16.5 (median) (13–21 days)	
Farquharson, A.L., 2011 [46]	Australia	RCT, DB	CABG or valve repair/replacement	I: 97	64 ± 11	80 (82%)	4.6 g/day as Liquid oil 15 mL/day EPA: 2.7, DHA: 1.9 g/day)	Not specified	3 week Pre-OP to Post-OD 6 or discharge 22 days (median)	(-) Length of time in cardiothoracic ICU (*p* = 0.006) (=) Total hospital length of stay (*p* = 0.24)
				C: 97	64 ± 10	62 (64%)	15 mL/day High monounsaturated sunflower oil		21 days (median)	
Sandesara, 2012 [48] (The FISH trial)	US	MC, DB, RCT	CABG with or without valve surgery	I: 120	63.4 ± 9.5	94 (78%)	Pre-operative: ≥4 g/day with minimum loading dose of 6 g over 2 days Post-operative: 2 g/day 465 mg EPA 375 mg DHA	1.24:1	Pre-OD 2 until occurrence of AF or 14 days	(=) hospital stay (*p* = 0.27) (=) ICU rehospitalization (*p* = 0.64)
				C: 123	62 ± 11.4	102 (83%)	Corn oil 2 g/day			
Farahani, 2017 [50]	Iran	SC, RCT, DB	CABG	I: 202	60.62 ± 8.95	132 (65%)	2 g/day fish oil 300 mg EPA 200 DHA	1.5:1	Pre-OD 5 to discharge	(-) ICU stay (*p* = 0.003) (-) Hospital stay (*p* = 0.04)
				C: 199	61.28 ± 10	127 (64%)	Olive oil soft gelatin capsules			
Sorice, M., 2011 [49]	Italy	RCT, DB	CABG “on-pump” and “off-pump”	I: 96	Off-pump 64 ± 10 On-pump 63 ± 10	Off-pump 37 (82%) On-pump: 39 (76%)	2 g/day	1:2	Pre-OD 5 to discharge	(=) Length of hospital stay (*p* = 0.75)
				C: 105	Off-pump (G1) 63 ± 10 On-pump (G2) 63 ± 9	G1) 42 (87%) (G2) 46 (80%)	No intervention			
OPERA 2012 (Mozaffarian) [45]	US, Italy, Argentina (28 centers)	Multinational RCT, DB	CABG, valve repair or replacement, other open cardiac surgery	I: 758	63.8 SD (12.6)	551 (72.7%)	Pre-operatively 10 g loading over 3–5 days or 8 g over 2 days Post-operatively 2 g ≥840 g/capsule 465 mg EPA: 375 mg DHA	Not specified	Pre-OD 3–5 to Post-OD 10 or discharge	(=) ICU stay (*p* = 0.38) (=) Hospital stay (*p* = 0.48)
				C: 758	63.6 SD (12.4)	543 (71.6%)	Olive oil			
Joss, 2017 [47]	US	RCT, DB	CABG, valve replacement/repair surgery	I: 284	66.6 ± 10.72	212 (74.7%)	2 g/day	2:3 DHA: EPA	Pre-OD 5 or 24 h Post-operatively to Post-operative week 4 75% of pts started and 25% post-OD1	(=) Length of hospital stay (*p* = 0.834)
				C: 275	66.4 ± 10.72	199 (72.4%)	Mineral oil with a trace amount of wheat germ oil			
Bernabe-Garcia, 2014 [51]	Mexico	RCT, DB	CABG	I: 12	58.7 ± 2.7	100%	2.4 g/day omega-3 in 4 capsules (1.6 g/day EPA + 0.8 g DHA)	Not specified	Pre-OD 1 to Post-OD 6	(=) ICU stay, days (*p*= 0.440) (-) Total hospital stays, days (*p* = 0.038)
				C: 11	66.0 ± 2.5	100%	Corn starch			

Abbreviations: RCT: randomized controlled trial, DB: double-blind, PC: placebo-control, SB: single-blind, CPB: Cardiopulmonary Bypass, ICU: intensive care unit; OL: open-label, SC: single-center, MC: multicenter, CABG: coronary artery bypass graft, F: female, M: male, Pre-OD: pre-operative day, Post-OD: post-operative day, FFA: free fatty acids, FO: fish oil, CCM: continuous cardiac monitor, DHA: Docosahexaenoic acid, EPA: Eicosapentaenoic acid, AF: Atrial fibrillation. * *p*-value not present, significance is narratively reported. (-) represent reduction effect. (=) represent no effect.

#### 3.2.1. IV Administration

The IV administration of omega-3 was at 12 or 24 h pre-operatively—a very short time when compared to oral supplementation—and continued until the first post-operative day, or second to seventh POD, or until discharge [29,42,43]. The dose ranged from 100 mg/kg to 400 mg/kg. The ratio of EPA to DHA was not specified in studies.

#### 3.2.2. Oral Supplementation

Nine studies introduced omega-3 PUFA by oral supplementation. The lowest dose provided was 1.7 g/day provided by Calò [52]. Four studies provided a dose of approximately 2 g/day of omega-3 capsules [44,47,49,50]. One study administered 2.4 g/day of omega-3 [51]. Two studies provided omega-3 loading of 6 g over 2 days pre-operatively and a 10 g loading dose over 3–5 days pre-operatively [45,48]. One study provided the highest dose of omega-3 PUFA of 4.6 g/day in a liquid form of 15 mL/day [46]. The duration of supplementation ranged from a median of 17 days pre-operatively to 4 weeks post-operatively or discharge. The most common duration of supplementation was 5 days pre-operatively to discharge [45,49,50,52]. There were discrepancies regarding the ratio of EPA to DHA among studies. Two studies used 1:2 ratios [49,52]. Two provided (1.2:1) [44,48], while the rest provided (2:3) [47] and (1.5:1) [50] EPA to DHA ratios. Finally, three studies did not report the EPA to DHA ratio [45,46,51].

### 3.3. Internal Validity (Risk-of-Bias Assessment)

The overall judgment of the twelve included studies was a low risk of bias in five (41.6%) [43,44,45,46,50], some concerns in three (25%) [29,47,51], and a high risk of bias was seen in four (33.3%) [42,48,49,52].

In the domain of bias due to randomization, most of the studies (75%) reported that it was randomized, or in computer-generated randomization the risk was assessed as low [29,43,44,45,46,48,50,51,52]. One trial did not provide details about the randomization process [42] and another had a significant difference in the baseline characteristics table [47], giving them “some concerns” as a judgment. The allocation was not concealed in the study [49] which increased the risk of bias in their paper.

**Figure 2:**
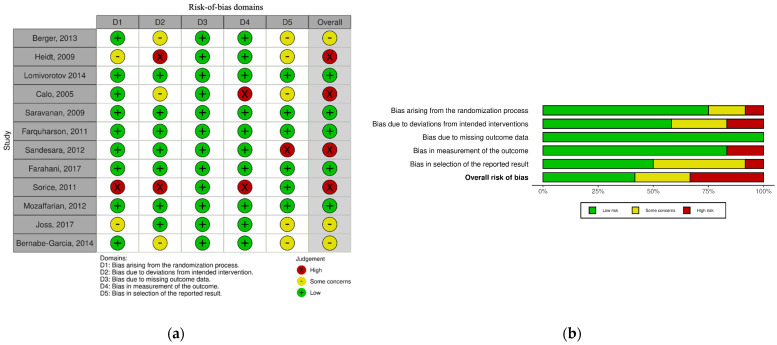
(**a**) Risk-of-bias summary of studies examining the effects of the omega-3 PUFA; (**b**) Risk-of-bias graph of studies examining the effects of the omega-3 PUFA. Done by according to Cochrane’s risk-of-bias tool for randomized trials [29,39,42,43,44,45,46,47,48,49,50,51,52].

Three papers had some concerns regarding bias arising from deviation from intended interventions [29,43,44,45,46,48,50,51,52], while two had a high risk of bias [42,49] in this domain. All of the papers had a low risk of bias regarding missing outcome data. In regard to bias due to the measurement of the outcome, the outcome assessors were aware of the intervention received by study participants which might influence the assessment of the outcome in [49,52], giving them the judgment of high risk of bias. Finally, when we assessed the bias in the selection of the reported result, five studies had some concerns [29,42,43,44,45,46,48,49,50,51,52] and one had a high risk of bias [48]. Figure 2a,b provide the risk-of-bias summary and graph of studies examining the effects of omega-3 PUFA.

### 3.4. External Validity

Regarding the external validity, most papers have a small sample size. However, the OPERA trial has a large sample size of 1516 participants and is a multinational study. Furthermore, Bernabe et al. [51] included only men in their study, and Berger et al. [29] included men only in the intervention arm, which makes them neither generalizable nor representative of the population. Moreover, some trials limited inclusion to only patients undergoing CABG by cardiopulmonary bypass (CPB) [43,44]. Based on this, the generalizability of these papers to the whole population of patients undergoing CABG surgery is questionable.

### 3.5. Source of Funding and Conflicts of Interest

There was heterogeneity among the studies concerning the sources of funding and conflict of interest disclosures. Five studies explicitly stated that authors had no conflicts of interest [29,42,47,50,51]. In contrast, five studies did not provide any conflict of interest disclosure [43,45,46,48,49,52]. Notably, only one study acknowledged the presence of conflicts of interest [44].

Regarding funding sources, five studies received support from pharmaceutical companies [44,45,47,48,50]. Two studies declared no external funding [42,52], while three studies provided no information regarding funding sources [43,46,49].

### 3.6. Meta-Analysis (Quantitative Assessment)

#### Synthesis of Meta-Analysis

For the construction of the meta-analysis, effect measures of all included studies were extracted and summarized in Table S6. Only studies that reported means and standard deviations, or that could be estimated from normally distributed data, were included in the meta-analysis. The study by Heidt et al. was excluded from the meta-analysis because it compared ICU and hospital stay between patients who developed atrial fibrillation and those who did not, without assessing the effect of omega-3 on these outcomes.

Seven studies analyzed ICU length of stay after CABG surgery. Four studies reported mean and standard deviation (SD) data and were directly included in the meta-analysis [29,43,46,51]. Three studies reported ICU length of stay as median and interquartile range (IQR). Their mean and SD were predicted; only one of them was eligible for inclusion in the meta-analysis [45]. Thus, the meta-analysis included five studies in total. It is important to note that there was heterogeneity in terms of sample size, omega-3 administration, dose, and duration. Table 2 summarizes included studies in the meta-analysis of the impact of omega-3 PUFA on the ICU length of stay outcome.

### 3.7. Forest Plot of the Effect of Omega-3 PUFA on ICU Stay

The meta-analysis for the length of intensive care unit stay for patients who had coronary artery bypass graft surgery was developed using the random effects model.

Overall, the administration of omega-3 long-chain fatty acids resulted in a tendency toward reduction in the length of stay in the intensive care unit compared to the control group, but no statistically significant effect was observed (mean difference and 95% confidence interval −0.25 [−0.68, 0.17]).

Subgroup analysis based on the method of administration was conducted and no effect was further seen on the duration of ICU stay, mean difference, and 95% confidence intervals (−0.30 [−0.99,0.39] and −0.59 [−1.7, 0.52] for intravenous and oral supplementation, respectively).

**Figure 3:**
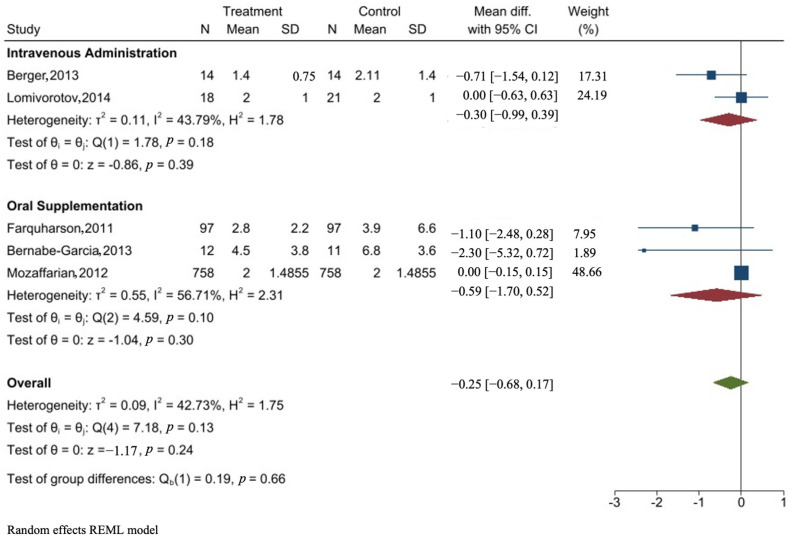
Meta-analysis and subgroup analysis of the impact of perioperative administration of omega-3 PUFA (intravenous and oral), compared to control (usual care or non-fish oils) on post-surgical intensive care unit stay in patients undergoing CABG surgery. The figure is done by STATA SE software version 17 [29,43,45,46,51].

The intravenous administration of omega-3 PUFA subgroup analysis included only two studies. There was a moderate heterogeneity between the two studies as per the I^2^ test equal to 43.79%. The dose administered was 200 mg/kg/day and 400 mg/kg/day of omega-3 in pre-operative day 1, and the post-operative duration was different between the studies. Both studies had small sample sizes, which make the power very small. In regard to the oral administration, there was substantial heterogeneity between the studies (I^2^ =56.71%). The study conducted by Mozaffarian et al. [45] had almost half the weight of the observed effect with 48.66%. Figure 3 shows the meta-analysis and subgroup analysis of the impact of perioperative administration of omega-3 PUFA on post-surgical intensive care unit stay in patients undergoing CABG surgery.

### 3.8. Assessment of Publication Bias

Publication bias was not assessed as the number of included studies in the meta-analysis was small.

#### 3.8.1. Effect of Omega-3 Polyunsaturated Fatty Acids (PUFA) on Hospital Length of Stay after Coronary Artery Bypass Graft (CABG) Surgery

Eleven studies analyzed hospital length of stay after CABG surgery, but only six studies were eligible for inclusion in the meta-analysis. Five studies reported mean and standard deviation (SD) data and were directly included in the meta-analysis [29,43,49,51,52]. The study by Sorice et al. (2011) [49] reported results in a subgroup based on the type of CABG surgery, so it was divided into two for constructing the meta-analysis. None of the studies that reported length of hospital stay as median and interquartile range (IQR) were eligible for inclusion in the meta-analysis after the prediction of their mean and standard deviation [44,45,48,50]. Other studies were also excluded as they were reported as adjusted mean ratio [46] and median and SD [47]. It is important to note that there was heterogeneity in terms of sample size, omega-3 administration, dose, and duration. Table 3 summarizes included studies in the meta-analysis of hospital length of stay outcomes after CABG surgery.

#### 3.8.2. Forest Plot of the Effect of Omega-3 PUFA on the Length of Hospital Stay

Overall, the meta-analysis showed that perioperative administration of omega-3 PUFA resulted in a significant reduction in the length of hospital stay for patients who underwent CABG surgery, with a mean difference (MD) of −0.58 days (95% confidence interval −1.13, −0.04).

**Figure 4:**
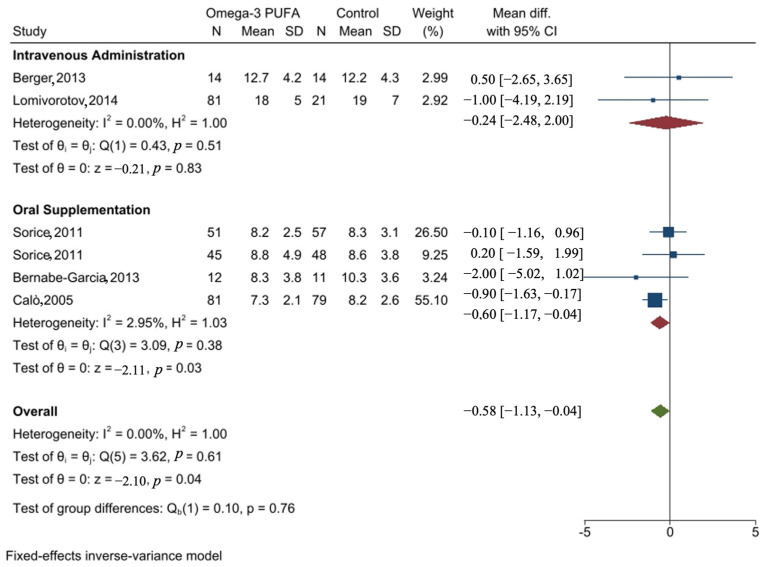
Meta-analysis and subgroup analysis of the impact of perioperative administration of omega-3 PUFA compared to control on hospital length of stay for patients undergoing CABG surgery. The figure is done by STATA SE software version 17 [29,43,49,51,52].

Subgroup analysis based on the method of administration revealed that intravenous administration of omega-3 PUFA did not result in any statistically significant difference in length of hospitalization (MD: −0.24 days; 95% CI: −2.48, 2.00). However, a significant reduction in the length of hospitalization was found in oral supplementation of the omega-3 PUFA subgroup (MD: −0.6 days; 95% CI: −1.17, −0.04). The study by Calò et al. (2005) [52] had the largest weight (55.1%), indicating that it had the greatest impact on the overall results. There was no significant heterogeneity between studies (I2 = 0%). Figure 4 shows the meta-analysis and subgroup analysis of the impact of perioperative administration of omega-3 PUFA compared to control on hospital length of stay for patients undergoing CABG surgery.

**Figure 5:**
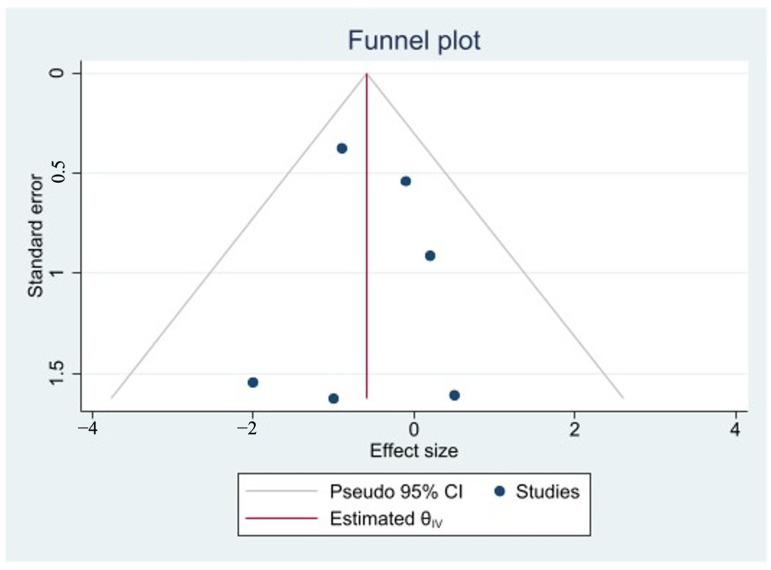
Funnel plot to assess publication bias for studies of length of hospital stay outcome.

Publication bias was assessed using Egger’s test and funnel plot analysis. Egger’s test showed no significant publication bias (*p* = 0.7531). Additionally, the funnel plot was symmetrical, indicating no evidence of publication bias. Figure 5 shows the funnel plot to assess publication bias for studies of length of hospital stay outcome.

## 4. Discussion

This systematic review provides an update of the current knowledge in regard to the effect of omega-3 PUFA supplementation on ICU stay and length of hospitalization post-CABG surgery. The systematic review is more concise to the effect of only omega-3 long-chain fatty acid (EPA + DHA) administration on clinical outcomes post-CABG surgery. Twelve studies have been identified for the qualitative synthesis and seven of them were eligible for inclusion in the meta-analysis.

The overall pooled mean difference between the omega-3 PUFA group and the control group regarding ICU stay after CABG surgery showed no statistical difference between the groups, but a tendency toward reduction was observed (−0.25 (95% CI −0.68, 0.17)). Subgroup analysis in terms of method of administration did not show any further significant effect (−0.30 (−0.99,0.39) and −0.59 (−1.7, 0.52)) for intravenous and oral supplementation, respectively.

Moreover, omega-3 PUFA was associated with a significant reduction in days of hospital stay (−0.58 (95% CI −1.13, −0.04)). Furthermore, subgroup analysis showed that oral supplementation resulted in a statistically significant reduction in length of hospitalization with MD (−0.6 (95% CI −1.17, −0.04)). However, no statistically significant difference in length of hospitalization was observed when omega-3 was administered intravenously (−0.24 (95% CI −2.48, 2.00)).

### 4.1. The Effect of Omega-3 PUFA on ICU Length of Stay Post-CABG Surgery

On average, there was no difference in the length of ICU stay between patients who received omega-3 polyunsaturated fatty acids (PUFAs) and those who did not after coronary artery bypass grafting (CABG) surgery (−0.25 (95% CI −0.68, 0.17)).

This finding is consistent with a previous systematic review, which found no effect of omega-3 PUFAs alone or combined with other vitamins on ICU length of stay in patients undergoing different cardiac surgeries (weighted mean difference −2.95 days, (95% CI −10.28, 4.39)) [34]. However, it is not consistent with another systematic review that found that administration of mainly antioxidant vitamin therapy—some with omega-3 PUFA—resulted in improvement in all clinical outcomes including intensive care unit length of stay (MD −0.21, 95% CI −0.30, −0.12, *p* < 0.00001) [35]. A possible explanation for the lack of effect on ICU stay could be that the anti-inflammatory effects are not potent enough to immediately reduce the post-operative inflammatory response after surgery but when combined with other vitamins, will produce a stronger effect and reduce ICU stay.

### 4.2. Omega-3 and Hospital Length of Stay Post-CABG Surgery

Omega-3 PUFA was associated with significant reduction in days of hospital stay (−0.58 (95% CI −1.13, −0.04)). A meta-analysis showed a significant effect of perioperative omega-3 PUFA on length of hospitalization post-cardiac surgeries [34]. Moreover, the perioperative use of antioxidant vitamin therapy resulted in a significant reduction of hospital stay duration (MD −0.68, 95% CI −0.98, −0.39, *p* < 0.00001). These findings are essentially consistent with the results of the current study. Reduction in length of hospitalization in response to perioperative omega-3 PUFA administration may be explained by several mechanisms.

First, the anti-inflammatory and pro-resolving effects of omega-3 PUFAs are likely to play a major role. One way that omega-3 PUFAs reduce inflammation is by competing with arachidonic acid for the production of eicosanoids. Eicosanoids are signaling molecules that play a role in a variety of physiological processes, including inflammation [53]. Omega-3 PUFAs, on the other hand, are converted to less pro-inflammatory eicosanoids.

Another way that omega-3 PUFAs reduce inflammation is by promoting the production of specialized pro-resolving mediators (SPMs) [53]. SPMs are a class of lipid mediators that are derived from EPA and DHA, including resolvins, protectins, and docosatrienes, and have potent anti-inflammatory and pro-resolving effects [54,55]. Enzymatic conversion of omega-3 polyunsaturated fatty acids into SPMs actively disrupts inflammatory circuits and skews the immune response toward repair and homeostasis [26,56,57,58].

The anti-inflammatory and pro-resolving effects of omega-3 PUFAs are likely to be particularly beneficial for patients who are at high risk for developing complications after surgery. For example, patients who undergo coronary artery bypass grafting (CABG) surgery are at high risk for developing post-operative inflammation, which can lead to a number of complications, such as arrhythmias, heart failure, and infection, as well as increasing length of hospitalization.

Furthermore, the antiarrhythmic effects of omega-3 PUFAs are likely to play a major role in reducing post-operative atrial fibrillation (POAF) and are thought to be mediated through a number of mechanisms, including modulation of ion channels and alteration of membrane phospholipid composition [59,60,61]. Omega-3 PUFAs can also alter the membrane phospholipid composition of cardiac cells [62]. This can lead to changes in the fluidity and permeability of the cell membrane, which can also affect the electrical activity of the heart and reduce the risk of arrhythmias [60].

Subgroup analysis showed that oral supplementation resulted in a statistically significant reduction in length of MD (−0.6 (95% CI −1.17, −0.04)). However, no statistically significant difference in length of hospitalization was observed when omega-3 was administered intravenously (−0.24 (95% CI −2.48, 2.00)). The observed effect might be due to the fact that oral supplementation has a longer duration compared to the intravenously administered omega-3 PUFA, resulting in the accumulation of more omega-3 PUFAs in the body and better anti-inflammatory functions.

Regarding the risk of bias and external validity, the studies differed in terms of bias and most of the papers have small sample sizes; some trials limited inclusion to only patients undergoing only CABG by cardiopulmonary bypass (CPB). Based on this, the samples for some trials are not representative of the target population, which makes them not generalizable to the population.

### 4.3. Implications for Future Research

The findings of this systematic review and meta-analysis suggest that omega-3 PUFA supplementation, particularly oral administration, may be an effective intervention for reducing the length of hospitalization following CABG surgery. The potential benefits of omega-3 PUFAs are likely mediated by their anti-inflammatory, pro-resolving, and antiarrhythmic properties. Further research is warranted to optimize the dose, duration, and formulation of omega-3 PUFA supplementation for maximizing clinical benefits in the perioperative setting.

### 4.4. Strengths and Limitations

This systematic review and meta-analysis has several strengths: it is an up-to-date and comprehensive assessment of the effects of omega-3 PUFA on post-CABG ICU and hospital stay, incorporating two new studies published since the last review in 2017 [34]. Unlike previous analyses that included various surgeries, this review focuses solely on CABG surgery to provide more relevant insights. A thorough search strategy was applied, covering multiple databases with a comprehensive list of key terms up to 12 September 2023, ensuring no relevant studies were missed. Only randomized controlled trials were included, with most being double-blinded, enhancing the internal validity of the findings. Additionally, two independent reviewers assessed study inclusion and risk of bias, ensuring objectivity. The review also uniquely evaluates external validity, funding sources, and conflicts of interest, further strengthening the reliability and broader applicability of the findings.

The current systematic review also has several limitations. The evidence provided by this review was based on a relatively small sample size for most papers (<100 participants), which might affect overall power and restrict the generalizability of the studies. Furthermore, the included studies did not all have good methodological quality, there was heterogeneity between studies, and two open-label trials were added to the review, which may weaken the strength of the conclusion. Furthermore, the heterogeneity between studies in their populations, omega-3 dose, and duration of supplementation makes it difficult to compare results and to detect the true effect. Finally, not all studies are included in the meta-analysis due to heterogeneity in effect measures, and the largest study was not included in the length of hospital stay meta-analysis.

## 5. Conclusions

This updated systematic review and meta-analysis both suggest that perioperative omega-3 PUFA administered orally may be associated with improvements in hospitalization length, potentially due to anti-inflammatory and antiarrhythmic effects. However, no significant benefit was observed regarding ICU stay reduction. The heterogeneity and generally low quality of the included studies warrant cautious interpretation of these findings. Further rigorous, large-scale studies are needed to substantiate these potential benefits.

## Figures and Tables

**Table 2:** Summary of included studies in meta-analysis of the impact of omega-3 PUFA on intensive care unit (ICU) stay.

Study	Method of Administration	Dose	Pre-Operative	Post-Operative	Mean Difference	95% Confidence Interval
Berger, 2013 [29]	IV	400 mg	Day 1	Day 1	−0.71	[−1.54, 0.12]
Lomivorotov, 2014 [43]	IV	Pre-OP 200 mg Post-OP 100 mg	Day 1	Days 2–7	0.00	[−0.63, 0.63]
Farquharson, 2011 [46]	Oral	4.6 g/day	3 weeks	Day 6	−1.10	[−2.48, 0.28]
Bernabe-Garcia, 2014 [51]	Oral	2.4 g/day	Day 1	Day 6	−2.30	[−5.32, 0.72]
Mozaffarian, 2012 [45]	Oral	8 g over 2 day Pre-OP 2 g Post-OP	Days 3–5	Day 10 or Discharge	0.00	[−0.15, 0.15]

**Table 3:** Summary of included studies in meta-analysis of the impact of omega-3 PUFA on hospital length of stay after CABG surgery.

Study	Method of Administration	Dose	Pre-Operative	Post-Operative	Mean Difference	95% Confidence Interval
Berger, 2013 [29]	IV	400 mg	Day 1	Day 1	0.5	[−2.65, 3.65]
Lomivorotov, 2014 [43]	IV	Pre-OP 200 mg Post-OP 100 mg	Day 1	Days 2–7	−1	[−4.19, 2.19]
Sorice, 2011 [49] “on-pump CABG”	Oral	2 g/day	Day 5	Discharge	−0.1	[−1.16, 0.96]
Sorice, 2011 “on-pump CABG” [49]	Oral	2 g/day	Day 5	Discharge	0.2	[−1.59, 1.99]
Bernabe-Garcia, 2014 [51]	Oral	2.4 g/day	Day 1	Day 6	−2	[−5.02, 1.02]
Calò, 2005 [52]	Oral	1.7 g/day	Day 5	Discharge	−0.9	[−1.63, −0.17]

## Data Availability

All data generated or analyzed during this study are included in this article. Further inquiries can be directed to the corresponding author.

## Supplementary Materials

The following supporting information can be downloaded at https://www.mdpi.com/article/10.3390/nu16193298/s1: Table S1: Prisma 2020 checklist; Table S2: Search strategy table from Medine, EMBASE, CINAHL, PsycINFO, and the Cochrane Central Register of Controlled Trial Library; Table S3: Detailed search strategy for EMBASE database; Table S4: Excluded studies after full text screening from electronic databases; Table S5: Table of Excluded studies after full text screening from Supplementary; Table S6: Summary table of all effect measures extracted from included studies for the construction of the meta-analysis.
