# Supplementary material for: Omega-3 Supplementation in Coronary Artery Bypass Graft Patients: Impact on ICU Stay and Hospital Stay—A Systematic Review and Meta-Analysis
Source: Nutrients. 2024 Sep 29;16(19):3298. doi: 10.3390/nu16193298 (PMC11478518; doi:10.3390/nu16193298)
Supplement: Supplementary file 1 [file nutrients-16-03298-s001.zip › nutrients-3176830-supplementary.pdf]

Supplementary Materials

Table **S1**. Prisma 2020 checklist

Section and Topic	Item #	Checklist item	Location where item is reported
**TITLE**			
Title	1	Identify the report as a systematic review.	iv
**ABSTRACT**			
Abstract	2	See the PRISMA 2020 for Abstracts checklist.	iv
**INTRODUCTION**			
Rationale	3	Describe the rationale for the review in the context of existing knowledge.	1- 2
Objectives	4	Provide an explicit statement of the objective(s) or question(s) the review addresses.	2
**METHODS**			
Eligibility criteria	5	Specify the inclusion and exclusion criteria for the review and how studies were grouped for the syntheses.	17
Information sources	6	Specify all databases, registers, websites, organisations, reference lists and other sources searched or consulted to identify studies. Specify the date when each source was last searched or consulted.	15-16
Search strategy	7	Present the full search strategies for all databases, registers and websites, including any filters and limits used.	16. Appendix B&C
Selection process	8	Specify the methods used to decide whether a study met the inclusion criteria of the review, including how many reviewers screened each record and each report retrieved, whether they worked independently, and if applicable, details of automation tools used in the process.	17
Data collection process	9	Specify the methods used to collect data from reports, including how many reviewers collected data from each report, whether they worked independently, any processes for obtaining or confirming data from study investigators, and if applicable, details of automation tools used in the process.	18
Data items	10a	List and define all outcomes for which data were sought. Specify whether all results that were compatible with each outcome domain in each study were sought (e.g. for all measures, time points, analyses), and if not, the methods used to decide which results to collect.	18
	10b	List and define all other variables for which data were sought (e.g. participant and intervention characteristics, funding sources). Describe any assumptions made about any missing or unclear information.	18
Study risk of bias assessment	11	Specify the methods used to assess risk of bias in the included studies, including details of the tool(s) used, how many reviewers assessed each study and whether they worked independently, and if applicable, details of automation tools used in the process.	18

Section and Topic	Item #	Checklist item	Location where item is reported
Effect measures	12	Specify for each outcome the effect measure(s) (e.g. risk ratio, mean difference) used in the synthesis or presentation of results.	20
Synthesis methods	13a	Describe the processes used to decide which studies were eligible for each synthesis (e.g. tabulating the study intervention characteristics and comparing against the planned groups for each synthesis (item #5)).	19
	13b	Describe any methods required to prepare the data for presentation or synthesis, such as handling of missing summary statistics, or data conversions.	19
	13c	Describe any methods used to tabulate or visually display results of individual studies and syntheses.	19
	13d	Describe any methods used to synthesize results and provide a rationale for the choice(s). If meta-analysis was performed, describe the model(s), method(s) to identify the presence and extent of statistical heterogeneity, and software package(s) used.	20
	13e	Describe any methods used to explore possible causes of heterogeneity among study results (e.g. subgroup analysis, meta-regression).	19
	13f	Describe any sensitivity analyses conducted to assess robustness of the synthesized results.	19
Reporting bias assessment	14	Describe any methods used to assess risk of bias due to missing results in a synthesis (arising from reporting biases).	18
Certainty assessment	15	Describe any methods used to assess certainty (or confidence) in the body of evidence for an outcome.	Not reported
**RESULTS**			
Study selection	16a	Describe the results of the search and selection process, from the number of records identified in the search to the number of studies included in the review, ideally using a flow diagram.	21-22
	16b	Cite studies that might appear to meet the inclusion criteria, but which were excluded, and explain why they were excluded.	Appendix D&E
Study characteristics	17	Cite each included study and present its characteristics.	26-30
Risk of bias in studies	18	Present assessments of risk of bias for each included study.	32
Results of individual studies	19	For all outcomes, present, for each study: (a) summary statistics for each group (where appropriate) and (b) an effect estimate and its precision (e.g. confidence/credible interval), ideally using structured tables or plots.	35,38
Results of syntheses	20a	For each synthesis, briefly summarise the characteristics and risk of bias among contributing studies.	23-25, 31
	20b	Present results of all statistical syntheses conducted. If meta-analysis was done, present for each the summary estimate and its precision (e.g. confidence/credible interval) and measures of statistical heterogeneity. If comparing groups, describe the direction of the effect.	37-40

Section and Topic	Item #	Checklist item	Location where item is reported
	20c	Present results of all investigations of possible causes of heterogeneity among study results.	38,39
	20d	Present results of all sensitivity analyses conducted to assess the robustness of the synthesized results.	37-40
Reporting biases	21	Present assessments of risk of bias due to missing results (arising from reporting biases) for each synthesis assessed.	Not Reported
Certainty of evidence	22	Present assessments of certainty (or confidence) in the body of evidence for each outcome assessed.	Not Reported
**DISCUSSION**			
Discussion	23a	Provide a general interpretation of the results in the context of other evidence.	42
	23b	Discuss any limitations of the evidence included in the review.	46
	23c	Discuss any limitations of the review processes used.	46
	23d	Discuss implications of the results for practice, policy, and future research.	45
**OTHER INFORMATION**			
Registration and protocol	24a	Provide registration information for the review, including register name and registration number, or state that the review was not registered.	15
	24b	Indicate where the review protocol can be accessed, or state that a protocol was not prepared.	15
	24c	Describe and explain any amendments to information provided at registration or in the protocol.	15
Support	25	Describe sources of financial or non-financial support for the review, and the role of the funders or sponsors in the review.	NA
Competing interests	26	Declare any competing interests of review authors.	NA
Availability of data, code and other materials	27	Report which of the following are publicly available and where they can be found: template data collection forms; data extracted from included studies; data used for all analyses; analytic code; any other materials used in the review.	NA

From: Page MJ, McKenzie JE, Bossuyt PM, Boutron I, Hoffmann TC, Mulrow CD, et al. The PRISMA 2020 statement: an updated guideline for reporting systematic reviews. BMJ 2021;372:n71. doi: 10.1136/bmj.n71

Table **S2**. Search strategy table from Medine, EMBASE, CINAHL, PsycINFO and Cochrane Central Register of Controlled Trial

**Database**	**Fatty Acids**	**Coronary Artery Bypass**	**ICU Stay**	**Length of Hospital Stay**
Medline	exp *Fatty Acids, Omega-3/<br>“Omega-3”<br>“Omega 3”<br>“Fish oil”<br>"Eicosapentaenoic Acid"<br>"Eicosapentenoic Acid"<br>EPA<br>"Docosahexaenoic acid*"<br>"Docosahexenoic acid*"<br>DHA<br>n3<br>n-3<br>“Alphalinolen* acid”<br>“Alpha-linolen* acid”	exp *Coronary artery bypass/<br>“aortocoronary Bypass”<br>“coronary artery bypass”<br>CABG	Intensive care unit/<br><br>“Intensive Care Unit”<br>“Unit, Intensive Care”<br>“ICU Intensive Care Units”	Length of stay/<br><br>“Stay Length”<br>“Stay Lengths”<br>“Hospital Stay”<br>“Hospital Stays”<br>“Stay, Hospital”<br>“Stays, Hospital”
EMBASE	omega 3 fatty acid/<br>“Omega-3”<br>“Omega 3”<br>“Fish oil”<br>"Eicosapentaenoic Acid"<br>"Eicosapentenoic Acid"<br>EPA<br>"Docosahexaenoic acid*"<br>"Docosahexenoic acid*"<br>DHA<br>n3<br>n-3<br>“Alphalinolen* acid”<br>“Alpha-linolen* acid”	exp *coronary artery bypass graft/<br>“aortocoronary Bypass”<br>“coronary artery bypass”<br>CABG	Exp *intensive care unit/<br>“close attention unit”<br>“combined medical and surgical icu”<br>“intensive care”<br>“cardiovascular icu”<br>“coronary care units” “coronary intensive care unit”	exp *hospitalization/<br><br>'”length of hospital stay”<br>“hospital stay”<br>“hospitalization”<br>“short stay” hospitalization”
PsychINFO	Not relevant	Not relevant	Not relevant	Not relevant
CINAHL	exp *Fatty Acids, Omega-3/<br>“Omega-3”<br>“Omega 3”<br>“Fish oil”	exp *Coronary artery bypass/<br>“aortocoronary Bypass”<br>“coronary artery bypass”<br>CABG	Exp *intensive care unit/<br>“close attention unit”<br>“'medical/surgical icus”<br>“mixed medical and surgical icu”	exp *hospitalization/<br><br>'”length of hospital stay”<br>“hospital stay”

	"Eicosapentaenoic Acid"<br>"Eicosapentenoic Acid"<br>EPA<br>"Docosahexaenoic acid*"<br>"Docosahexenoic acid*"<br>DHA<br>n3<br>n-3<br>“Alphalinolen* acid”<br>“Alpha-linolen* acid”		“mixed surgical and medical icu”<br>“respiratory care unit”<br>“respiratory care units” “special care unit”	“hospitalization”<br>“short stay” hospitalization”
Cochrane Central Register of Controlled Trial	exp *Fatty Acids, Omega-3/<br>“Omega-3”<br>“Omega 3”<br>“Fish oil”<br>"Eicosapentaenoic Acid"<br>"Eicosapentenoic Acid"<br>EPA<br>"Docosahexaenoic acid*"<br>"Docosahexenoic acid*"<br>DHA<br>n3, n-3<br>“Alphalinolen* acid”<br>“Alpha-linolen* acid”	exp *Coronary artery bypass/<br>“aortocoronary Bypass”<br>“coronary artery bypass”<br>CABG	Exp *intensive care unit/<br>“ICU stay”<br>“Intensive care unit stay”<br>“close attention unit”<br>“intensive care”<br>“mixed surgical and medical icu”<br>“coronary care units” “coronary intensive care unit”	exp *hospitalization/<br>“ Hospitalization duration”<br>”length of hospital stay”<br>“hospital stay”<br>“hospitalization”

Table **S3**. Detailed search strategy for EMBASE database

('coronary artery bypass graft'/exp OR 'aorta coronary artery bypass' OR 'aorta coronary bypass' OR 'aorta coronary bypass graft' OR 'aorta coronary vein bypass' OR 'aorta coronary vein bypass graft' OR 'aorta coronary vein shunt' OR 'aortic coronary artery bypass' OR 'aortic coronary bypass' OR 'aorticocoronary anastomosis' OR 'aorto coronary artery bypass' OR 'aorto coronary bypass graft' OR 'aorto coronary vein bypass' OR 'aortocoronary anastomosis' OR 'aortocoronary artery bypass' OR 'aortocoronary artery bypass graft' OR 'aortocoronary bypass' OR 'aortocoronary bypass graft' OR 'aortocoronary shunt' OR 'aortocoronary vein bypass' OR 'aortocoronary vein bypass graft' OR 'aortocoronary venous bypass' OR 'aortocoronary venous bypass graft' OR 'coronary artery bypass' OR 'coronary artery bypass graft' OR 'coronary artery bypass grafting' OR 'coronary artery graft' OR 'coronary bypass' OR 'coronary bypass graft' OR 'coronary bypass grafting' OR 'coronary vein bypass graft' OR 'coronary venous bypass graft' OR 'heart surgery'/exp OR 'cardiac surgery' OR 'cardiac surgical procedures' OR 'cardiosurgery' OR 'heart operation' OR 'heart surgery' OR 'myocardial resection' OR 'surgery, heart'

OR 'open heart surgery'/exp OR 'heart surgery, open' OR 'intracardiac surgery' OR 'open cardiac surgery' OR 'open heart surgery' OR cabg OR 'cabg surgery') AND ('omega 3 fatty acid'/exp OR 'bilantin omega' OR 'conchol 36' OR 'eicosa e' OR 'eicosapen' OR 'fatty acids, omega 3' OR 'fatty acids, omega-3' OR 'n 3 fatty acid' OR 'n 3 polyunsaturated fatty acid' OR 'omega 3' OR 'omega 3 carboxylic acid' OR 'omega 3 carboxylic acids' OR 'omega 3 fatty acid' OR 'omega 3 feingold' OR 'omega 3 plus' OR 'omega 3 polyunsaturated fatty acid' OR 'omega forte' OR 'omega-3-carboxylic acids' OR 'omega3 polyunsaturated fatty acid' OR 'sanhelios omega 3' OR 'fish oil'/exp OR 'fish oil' OR 'fish oils' OR 'omegaven' OR 'optimepa' OR 'tuna oil' OR 'marine oil'/exp OR 'eicosapentanoic acid'/exp OR 'lipid emulsion'/exp OR 'emulsion, fat' OR 'emulsion, lipid' OR 'fat emulsion' OR 'fat emulsions, intravenous' OR 'lipid emulsion' OR 'icosapentaenoic acid'/exp OR '5, 8, 11, 14, 17 eicosapentaenoic acid' OR '5, 8, 11, 14, 17 icosapentaenoic acid' OR '5, 8, 11, 14, 17-eicosapentaenoic acid' OR 'eicosa 5, 8, 11, 14, 17 pentaene carboxylic acid' OR 'eicosa 5, 8, 11, 14, 17 pentaenoic acid' OR 'eicosapentaenoate' OR 'eicosapentaenoic acid' OR 'eicosapentenoic acid' OR 'epaspire' OR 'icosa 5, 8, 11, 14, 17 pentaenoic acid' OR 'icosapent' OR 'icosapentaenoate' OR 'icosapentaenoic acid' OR 'omega 3 eicosapentaenoic acid' OR 'timnodonate' OR 'timnodonic acid' OR 'docosahexaenoic acid'/exp OR 'dhasco' OR 'docosahexaenoate' OR 'docosahexaenoic acid' OR 'docosahexaenoic acids' OR 'docosahexenoic acid' OR 'n 3'/exp) AND ('length of hospital stay' OR 'hospitalization'/exp OR 'hospital stay' OR 'hospitalization' OR 'short stay hospitalization' OR 'intensive care unit'/exp OR 'gicu' OR 'gicus' OR 'icu`s' OR 'close attention unit' OR 'combined medical and surgical icu' OR 'combined surgical and medical icu' OR 'critical care unit' OR 'general icu' OR 'intensive care department' OR 'intensive care unit' OR 'intensive care units' OR 'intensive therapy unit' OR 'intensive treatment unit' OR 'medical-surgery icu' OR 'medical/surgical icu' OR 'medical/surgical icus' OR 'medico-surgical icu' OR 'mixed medical and surgical icu' OR 'mixed surgical and medical icu' OR 'respiratory care unit' OR 'respiratory care units' OR 'special care unit' OR 'surgery/medical icu' OR 'surgical-medical icus' OR 'surgical/medical icu' OR 'unit, intensive care' OR 'cardiac surgery intensive care unit'/exp OR 'surgical intensive care unit'/exp OR 'hospitalization length of stay' OR 'coronary care unit'/exp OR 'cardiac icu' OR 'cardiac icus' OR 'cardiac intensive care unit' OR 'cardio-vascular intensive care unit' OR 'cardiologic unit' OR 'cardiology icu' OR 'cardiology intensive care unit' OR 'cardiology unit' OR 'cardiovascular icu' OR 'cardiovascular intensive care unit' OR 'coronary icu' OR 'coronary care unit' OR 'coronary care units' OR 'coronary intensive care unit' OR 'coronary resuscitation unit' OR 'coronary unit')

Table **S4**. Table of Excluded studies after full text-screening from electronic databases

Title	Reason for exclusion
**Does treatment with n-3 polyunsaturated fatty acids prevent atrial fibrillation after open heart surgery?**	population
**Effect of dietary supplementation with n-3 fatty acids on coronary artery bypass graft patency**	Outcome
**Antioxidant Supplementation Attenuates Oxidative Stress in Patients Undergoing Coronary Artery Bypass Graft Surgery**	Outcome
**Effect of pretreatment with omega-3 polyunsaturated fatty acids (PUfas) on hematological parameters and platelets aggregation in patients during elective coronary artery bypass grafting**	outcome
**Myocardial protection during elective coronary artery bypasses grafting by pretreatment with omega-3 polyunsaturated fatty acids**	Outcome
**Plasma n-3 and n-6 fatty acids and the incidence of atrial fibrillation following coronary artery bypass graft surgery**	Study design

**Omega-3 fatty acids do not alter P-wave parameters in electrocardiogram or expression of atrial connexins in patients undergoing coronary artery bypass surgery**	Outcome
**Vascular prostacyclin is increased in patients ingesting omega-3 polyunsaturated fatty acids before coronary artery bypass graft surgery**	Outcome
**Preoperative carbohydrate load and intraoperatively infused omega-3 polyunsaturated fatty acids positively impact nosocomial morbidity after coronary artery bypass grafting: a double-blind controlled randomized trial**	Exposure
**Prevention The effect of omega-3 polyunsaturated fatty acids in prevention of postoperative atrial fibrillation development in patients undergoing coronary artery bypass graft surgery**	study design
**Marine n-3 fatty acids are incorporated into atrial tissue but do not correlate with postoperative atrial fibrillation in cardiac surgery**	study design
**A small cohort omega-3 PUFA supplement study: implications of stratifying according to lipid membrane incorporation in cardiac surgical patients**	study design

Appendix SA. Table of Excluded studies after full text-screening from Supplementary Search

Title	Reason for exclusion
**A small cohort omega-3 PUFA supplement study: implications of stratifying according to lipid membrane incorporation in cardiac surgical patients**	Study design
**Preoperative n-3 polyunsaturated fatty acids are associated with a decrease in the incidence of early atrial fibrillation following cardiac surgery.**	Study design

**Prevention of new-onset atrial fibrillation after direct myocardial revascularization surgery: randomized comparative study**	Time of exposure-only post-operative period
**Omega-3 poly-unsaturated fatty acids reduce the incidence of postoperative atrial fibrillation in patients with history of prior myocardial infarction undergoing isolated coronary artery bypass grafting.**	Study design
**Protective effect of Eicosapentaenoic acid on insulin resistance in hyperlipidemic patients and on the postoperative course of cardiac surgery patients: the possible involvement of adiponectin.**	Exposure: only EPA molecule
**Effect of omega-3 polyunsaturated fatty acid on the prevention of atrial fibrillation after Off-pump coronary artery bypass grafting**	Study design

Table S6. Summary table of all effect measures extracted from included studies for the construction of Meta-analysis

Outcome	*Study*	*n*	*Mean*	*SD*	*n*	*Mean*	*SD*	*comment*
**ICU stay (days)**	Berger, M.M 2013	14	1.4	0.75	14	2.11	1.4	Included in MA
	farquharson 2011	97	2.8	2.2	97	3.9	6.6	
	Bernabe 2013	12	4.5	3.8	11	6.8	3.6	

	Lomivorotov 2014	18	2	1	21	2	1	
	Mozaffarian	758	7	2.9	758	6.6	2.2	Included in MA after calculation of mean and SD with sample normally distributed
	Study	n	Median	IQR	n	Median	IQR	
	Farahani 2017	202	2.4	(2.25-3)	199	1.54	(0.52-3.9)	Data are significantly skewed away from normality
	Mozaffarian	758	2	(1-3)	758	2	(1-3)	Included in MA, no significant evidence to show that the data are skewed
	saravanan	52	1	(1-2)	51	1	(1-2)	Data are significantly skewed away from normality
Outcome	***Study***	***n***	***Mean***	***SD***	***n***	***Mean***	***SD***	***Comment***
**Hospital Stay**	Berger, M.M 2013	14	12.7	4.2	14	12.2	4.3	Included in MA
	Calò 2005	81	7.3	2.1	79	8.2	2.6	
	Sorice (G2 n3, G4) on-pump	51	8.2	2.5	57	8.3	3.1	
	Sorice (G1 n3, G3) off-pump	45	8.8	4.9	48	8.6	3.8	
	Lomivorotov 2014	18	18	5	21	19	7	
	Bernabe 2013	12	8.3	3.8	11	10.3	3.6	
	Study	n	Adjusted mean ratio	SD	n	Adjusted mean ratio	SD	
	Farquharson 2011	97	8.6	7.1	97	9.9	10.2	Not included in MA
	Study	n	Median	IQR	n	Median	IQR	
	Farahani 2017(days)	202	14	(12-18)	199	14	(12-18)	the data are significantly skewed away from normality
	Mazaffarian (days)	758	7	(5-9)	758	7	(5-8)	the data are significantly skewed away from normality in intervention group

Sandesara, 2012 (Days)	120	6	(5-8)	123	5	(4-7)	the data are significantly skewed away from normality.
saravanan (days)	52	8.5	(6-12)	51	7	(6-10)	the data are significantly skewed away from normality in control group
Study	n	Median	SD	n	Median	SD	
Joss 2017 (days)	284	6	3.4	275	6	5	Not included in MA
